# Live and Let Die? Life Cycle Human Health Impacts from the Use of Tire Studs

**DOI:** 10.3390/ijerph15081774

**Published:** 2018-08-17

**Authors:** Anna Furberg, Rickard Arvidsson, Sverker Molander

**Affiliations:** Environmental Systems Analysis, Chalmers University of Technology, Gothenburg 412 96, Sweden; rickard.arvidsson@chalmers.se (R.A.); sverker.molander@chalmers.se (S.M.)

**Keywords:** life cycle assessment, LCA, disability-adjusted life years, DALY, Democratic Republic of the Congo, DRC, studded tires, passenger car

## Abstract

Studded tires are used in a number of countries during winter in order to prevent accidents. The use of tire studs is controversial and debated because of human health impacts from increased road particle emissions. The aims of this study are to assess whether the use of tire studs in a Scandinavian studded passenger car actually avoids or causes health impacts from a broader life cycle perspective, and to assess the distribution of these impacts over the life cycle. Life cycle assessment is applied and the disability-adjusted life years indicator is used to quantify the following five types of health impacts: (1) impacts saved in the use phase, (2) particle emissions in the use phase, (3) production system emissions, (4) occupational accidents in the production system, and (5) conflict casualties from revenues of cobalt mining. The results show that the health benefits in the use phase in general are outweighed by the negative impacts during the life cycle. The largest contribution to these negative human health impacts are from use phase particle emissions (67–77%) and occupational accidents during artisanal cobalt mining (8–18%). About 23–33% of the negative impacts occur outside Scandinavia, where the benefits occur. The results inform the current debate and highlight the need for research on alternatives to tire studs with a positive net health balance.

## 1. Introduction

Studded tires are used in many countries during winter, including Sweden, Norway, Finland, Russia, Canada, and the United States (U.S.) [1]. The function of the tire studs is to save lives by increasing the friction between the tires and the winter road, and thus reduce the number of accidents compared to if, for example, non-studded winter tires are being used [2]. At the same time, the use of studded tires is controversial and debated, as the release of worn road particles causes negative human health impacts [3]. In Sweden, the proponents of studded tires often raise the human health benefits of using tire studs. The opponents of studded tires on the other hand, mainly highlight the particle release as an argument for the introduction of restrictions. Furthermore, they also caution against the increased road pavement costs due to tire stud wear. Some countries have introduced restrictions of tire studs in passenger cars in the form of taxes or bans, such as Norway [3], leading to a 34% use of tire studs in passenger cars during winter [4]. In other countries, the studded tire use in passenger cars is notably higher (e.g., about 63% in Sweden [5] and 88% in Finland [6]).

The current debate about whether to use tire studs is generally limited to their use phase. However, for other products, a number of studies suggest a wider perspective for the assessment of human health impacts. Baumann et al. [7] assessed the human health impacts of an airbag system, including toxic life cycle emissions and direct impacts from accidents during metal mining, electricity production, and pyrotechnic material production, and compared these with the health impacts avoided in the use phase. That study could conclude that the use of an airbag system reduces health impacts more than its production causes. In the study by Gilbertson, et al. [8], human health impacts from the production of a nano-enabled chemical gas sensor were compared with its potential health benefits, showing a clear net benefit. In a study of a catalytic converter, the life cycle production impacts and use phase benefits in terms of reduced pollution were compared [9]. It was concluded that the product reduces or causes net health impacts depending on the value perspective chosen in the impact assessment method (i.e., a hierarchical or individualist versus an egalitarian). The study on the catalytic converter showed an uneven distribution of health impacts over the life cycle for some scenarios, where the negative health impacts of the people in countries where platinum-group metals are mined (South Africa and Russia) outweighed the positive health impacts from avoided air pollution in Sweden [9]. Another study quantified the negative human health impacts of a golden ring, including impacts from environmental emissions, occupational accidents, and impacts from the conflict in the Democratic Republic of the Congo (DRC), which is financed partly from gold mining [10]. The results showed considerable health impacts for the golden ring, in particular from the DRC conflict. Kobayashi et al. [11] applied a life cycle perspective for assessing the net health impacts of infrastructure for air pollution emission reduction in order to detect potential problem shifting of human health impacts in air quality policy making. The results indicated that improving local air quality would indeed cause a shift to more health impacts elsewhere.

All of these studies, to various extents, widen the perspective from human health impacts in the use phase of products to also consider the life cycle impacts and their distribution over the life cycle. To the knowledge of the authors, no such wider assessment has so far been conducted for tire studs. In a similar spirit to these other studies, the aims of this study are (i) to assess whether the use of the tire studs in a Scandinavian studded passenger car avoids or causes health impacts by applying a broader life cycle perspective, and (ii) to investigate the distribution of health impacts over the life cycle. The results provide information to decision makers and widen the current debate on the human health benefits and impacts from the use of studded tires.

## 2. Materials and Methods

A cradle-to-grave attributional life cycle assessment (LCA) was conducted, meaning that the whole tire stud life cycle was included and that the impacts associated with the entire product system (rather than impacts of changes in the product system) were quantified [12,13]. The function of tire studs is to save lives by increasing the grip between the tires and the road, and thus reduce the number of accidents. The functional unit was set to the weight of the tire studs in one passenger car with four studded tires. One tire stud consists of a 0.2–0.4 g pin made out of tungsten carbide with cobalt (WC-Co), where the cobalt content is 6–10% and the number of tire studs in four studded tires varies between about 480–540 [14]. The pin is surrounded by a 0.85 g aluminum body [15]. Thus, the functional unit corresponds to 0.10–0.22 kg of WC-Co and 0.40–0.46 kg aluminum.

The impact assessment was based on the method for assessing human health impacts developed by Arvidsson et al. [16], in which different positive and negative contributions to human health from a product *X* are quantified and compared in terms of the disability-adjusted life years (DALY) indicator. Based on this, net human health impacts can be calculated according to the following:

(1) $${\mathrm{DALY}}_{X}=\sum_{p}{\mathrm{DALY}}_{p}-\sum_{n}{\mathrm{DALY}}_{n}$$

where *p* is the positive health impacts and *n* is the negative health impacts. DALY is a measure of the number of years lost because of premature death (YLL) and/or the number of years lost because of disability (YLD) [17]. DALY, which has been increasingly applied in LCA for the quantification of human health impacts [18], can also be applied to quantify the number of years saved because of the prevention of accidents that otherwise would have caused years lost. The YLD is calculated as follows:

(2) $${\mathrm{YLD}=I\times \mathrm{DW}}_{i}\times L$$

where *I* is the number of incidents (–), *DW_i_* is the disability weight with a value between 0 (perfect health) and 1 (death) (–), and *L* is the time of the disability until its severity changes or the person dies (year).

**Figure 1:**
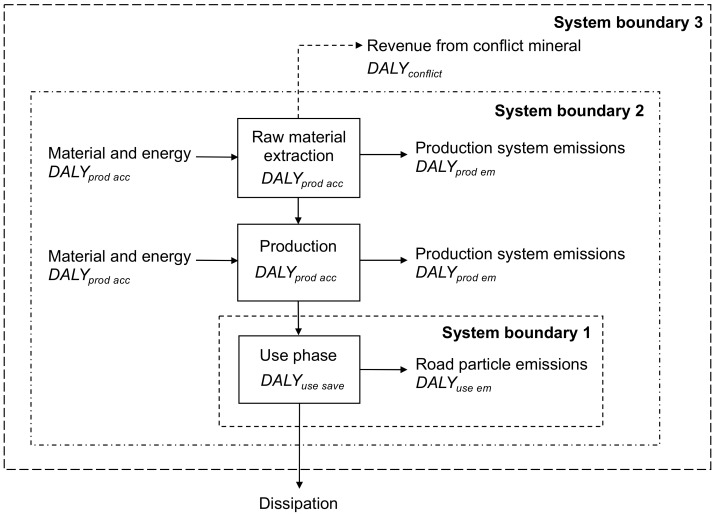
System studied and the three system boundaries considered. Physical flows are shown with solid arrows, and socio-economic influence with a dotted arrow. ${\mathrm{DALY}}_{\mathrm{use}\;\mathrm{save}}\;$= years saved by using studded tires instead of non-studded winter tires during winter; ${\mathrm{DALY}}_{\mathrm{use}\;\mathrm{em}}$ = years lost due to use phase emissions of road particles; ${\mathrm{DALY}}_{\mathrm{prod}\;\mathrm{em}}$ = years lost due to emissions in the production system of tire studs; ${\mathrm{DALY}}_{\mathrm{prod}\;\mathrm{acc}}$ = years lost due to accidents in the production system of tire studs; and ${\mathrm{DALY}}_{\mathrm{conflict}}$ = years lost due to revenues from cobalt mineral mining in the Democratic Republic of the Congo. DALY—disability-adjusted life years.

The net DALY from the use of tire studs in one passenger car was assessed by considering three system boundaries (Figure 1). System boundary 1 includes the use phase, which represents the perspective typically considered when human health impacts of studded tires are being discussed in the current debate. Positive health impacts occur from the avoidance of injuries in car crashes and the negative health impacts occur from the emissions of road particles. System boundary 2 expands the perspective from the use phase to the entire product life cycle of the tire studs, acknowledging that negative human health impacts are also caused by emissions and occupational accidents in the production of the raw materials and the tire studs. After the raw material extraction, production, and use phase of studded tires, the pins become dissipated [14], meaning that they are lost in a way that makes them unfeasible to recover, either by technical or economical means [19]. Thus, there is no specific waste management process for the tire studs [14]. At system boundary 3, the perspective is further expanded by considering also a socio-economic impact indirectly stemming from a physical flow in the product life cycle. The mining and trade of minerals are financing civil warfare in the DRC, which has gained international attention as reflected by, for example, the introduction of the Dodd–Frank act in 2010 [20]. This act defines tin, tantalum, tungsten, and gold as conflict minerals and puts requirements on publicly traded companies on the U.S. stock exchanges to report on their use of these minerals [20]. Other minerals mined in the DRC, including cobalt, have also been associated with the conflict [10]. Cobalt is mainly mined in the DRC, which is the number one cobalt producer in the world [21], and therefore, its contribution to negative human health impacts related to direct fatalities from the civil warfare were accounted for in this study. Contrary to cobalt, only a minor share (<1%) of the global tungsten production takes place in the DRC [22], which is why it was not included here.

The net DALY of the use of tire studs in one passenger car (${\mathrm{DALY}}_{\mathrm{tire}\;\mathrm{stud}}$) (year) was calculated according to the following operationalization on Equation (1)

(3) $${\mathrm{DALY}}_{\mathrm{tire}\;\mathrm{stud}}={\mathrm{DALY}}_{\mathrm{use}\;\mathrm{save}}-{\mathrm{DALY}}_{\mathrm{use}\;\mathrm{em}}-{\mathrm{DALY}}_{\mathrm{prod}\;\mathrm{em}}-{\mathrm{DALY}}_{\mathrm{prod}\;\mathrm{acc}}-{\mathrm{DALY}}_{\mathrm{conflict}},$$

where ${\mathrm{DALY}}_{\mathrm{use}\;\mathrm{save}}$ is the DALY saved by using studded tires instead of non-studded winter tires during winter (year), ${\mathrm{DALY}}_{\mathrm{use}\;\mathrm{em}}$ is the DALY lost due to emissions of particles in the use phase (year), ${\mathrm{DALY}}_{\mathrm{prod}\;\mathrm{em}}$ is the DALY lost due to emissions from the production system of tire studs (year), ${\mathrm{DALY}}_{\mathrm{prod}\;\mathrm{acc}}$ is the DALY lost due to accidents occurring in the production system (year), and ${\mathrm{DALY}}_{\mathrm{conflict}}$ is the DALY lost due to conflicts financed by revenues from the cobalt mined in the DRC (year). The system boundary 1 includes ${\mathrm{DALY}}_{\mathrm{use}\;\mathrm{save}}$ and ${\mathrm{DALY}}_{\mathrm{use}\;\mathrm{em}}$. The additional factors of ${\mathrm{DALY}}_{\mathrm{prod}\;\mathrm{em}}$ and ${\mathrm{DALY}}_{\mathrm{prod}\;\mathrm{acc}}$ are considered within system boundary 2. In system boundary 3, the perspective is further expanded to also include the ${\mathrm{DALY}}_{\mathrm{conflict}}$.

Data was selected to represent the current situation in Scandinavian countries (i.e., Sweden, Norway, and Denmark), where studded tires are frequently used and for which the necessary input data was available. Parameter uncertainties were considered by applying a low and high impact scenario for ${\mathrm{DALY}}_{\mathrm{tire}\;\mathrm{stud}}$, abbreviated LS and HS, respectively. When the annual data was available (e.g., as annual statistics), the lowest and highest value during the latest five-year period for which the data was available were selected. Detailed input data is presented in Section 2.1, Section 2.2, Section 2.3, Section 2.4, Section 2.5 and summarized in Tables S1 and S2 in the Supplementary Material (SM).

### 2.1. System Boundary 1: Lives Saved in the Use Phase

The DALY saved in the use phase of tire studs were calculated according to the following:

(4) $${\mathrm{DALY}}_{\mathrm{use}\;\mathrm{save}}=R\times N_{\mathrm{acc}}\times L_{\mathrm{tire}}\times {\mathrm{DALY}}_{\mathrm{car}\;\mathrm{acc}},$$

where R is the accident reduction rate that is expected if studded tires are used instead of non-studded winter tires (–), $N_{\mathrm{acc}}$ is the number of accidents that an average passenger car with non-studded winter tires is involved in during winter (accidents/year), $L_{\mathrm{tire}}$ is the lifetime of studded tires (year), and ${\mathrm{DALY}}_{\mathrm{car}\;\mathrm{acc}}$ is the number of DALY per car accident (year/accident).

Studded tires reduce passenger car accident rates by 2% on bare roads (assumed in the LS) and 5% on roads covered with ice or snow (assumed in the HS), compared with non-studded winter tires [2]. $N_{\mathrm{acc}}$ was derived from Norwegian statistics by dividing the number of accidents with passenger cars using non-studded winter tires during winter with the total number of such passenger cars, according to the following:

(5) $$N_{\mathrm{acc}}=\frac{N_{\mathrm{acc}\;\mathrm{tot}}}{N_{\mathrm{tot}\;\mathrm{car}}\times S_{\mathrm{car}\;\mathrm{non-studded}}},$$

where $N_{\mathrm{acc}\;\mathrm{tot}}$ is the total number of accidents with passenger cars using non-studded winter tires during winter (accidents/year), $N_{\mathrm{tot}\;\mathrm{car}}$ is the total number of registered passenger cars (–), and $S_{\mathrm{car}\;\mathrm{non-studded}}$ is the share of passenger cars with non-studded winter tires during winter (–). $N_{\mathrm{acc}\;\mathrm{tot}}$ was 860–1200 in the winters of 2012/2013–2016/2017 (November to April) in Norway [23]. $N_{\mathrm{tot}\;\mathrm{car}}$ was 2,400,000–2,700,000 [24], and the average $S_{\mathrm{car}\;\mathrm{non-studded}}$ was 46–64% [4], both for the years of 2012–2016 in Norway. Studded tires are used for 6–7 years on average [5].

The number of DALY lost per car accident was estimated by considering an average Swedish passenger car accident based on annual traffic statistics in 2013–2017 [25], as follows:

(6) $${\mathrm{DALY}}_{\mathrm{car}\;\mathrm{acc}}=\frac{N_{\mathrm{fatal}\;\mathrm{car}\;\mathrm{acc}}\times \left({\mathrm{LEX}}_{\mathrm{Sca}}-L_{\mathrm{death}}\right)+N_{\mathrm{severe}\;\mathrm{car}\;\mathrm{acc}}\times {\mathrm{DW}}_{\mathrm{severe}}\times L_{\mathrm{severe}}+N_{\mathrm{slight}\;\mathrm{car}\;\mathrm{acc}}\times {\mathrm{DW}}_{\mathrm{slight}}\times L_{\mathrm{slight}}\;}{N_{\mathrm{tot}\;\mathrm{car}\;\mathrm{acc}}},$$

where $N_{\mathrm{fatal}\;\mathrm{car}\;\mathrm{acc}}$ is the annual number of persons who lost their lives in fatal car accidents (–), ${\mathrm{LEX}}_{\mathrm{Sca}}$ is the life expectancy in Scandinavia (year), $L_{\mathrm{death}}$ is the age of the person at death (year), $N_{\mathrm{severe}\;\mathrm{car}\;\mathrm{acc}}$ is the annual number of persons who got severely injured in car accidents (–), ${\mathrm{DW}}_{\mathrm{severe}}$ is the disability weight for the severe injury (–), $L_{\mathrm{severe}}$ is the length of disability for the severe injury (year), $N_{\mathrm{slight}\;\mathrm{car}\;\mathrm{acc}}$ is the annual number of persons who got slightly injured in car accidents (–), ${\mathrm{DW}}_{\mathrm{slight}}$ is the disability weight for the slight injury (–), $L_{\mathrm{slight}}$ is the length of disability for the slight injury (year), and $N_{\mathrm{tot}\;\mathrm{car}\;\mathrm{acc}}$ is the total annual number of passenger car accidents (–). The number of persons in fatal, severe, and slight passenger car accidents, as well as the age information and the total number of passenger car accidents, were obtained from the Swedish government agency for Transport Analysis [25], and are as follows: in 2013–2017, 120–140 persons were killed in fatal accidents, 1300–1600 were severely injured, and 10,000–12,000 persons were slightly injured per year. The total number of passenger car accidents per year was approximately 9100–10,000 [25]. Here, ‘severe accidents’ imply, for example, fracture, crush injury, or concussion, while ‘slight accidents’ are other types of lower-impact injuries [26]. Based on this, a fracture in the hand, with disability weight 0.01, and fracture in the neck of femur, with disability weight 0.4 [27], were assumed for severe injuries in the LS and HS, respectively. The values for the disability weights of concussion and crush injury lie within this range. In the case of slight injuries, a sprain with the disability weight 0.008 was applied [27]. The length of disability for a fracture or sprain was based on the prognosis of recovery from such injuries, being about one to six months for fractures in general, and about two weeks to half a year for, for example, a sprained ankle [28]. ${\mathrm{LEX}}_{\mathrm{Sca}}$ was set to 81–82 years, which is the average Scandinavian life expectancy in 2012–2016 [29].

### 2.2. System Boundary 1: Particle Emissions in the Use Phase

Human health impacts from emissions of particles in the use phase of the tire studs were calculated according to the following:

(7) $${\mathrm{DALY}}_{\mathrm{use}\;\mathrm{em}}={\mathrm{CF}}_{\mathrm{PM}10\to \mathrm{DALY}}\times m_{\mathrm{road}\;\mathrm{particles}},$$

where ${\mathrm{CF}}_{\mathrm{PM}10\to \mathrm{DALY}}$ is an endpoint characterization factor converting emissions of PM_10_ to DALY (year/kg PM_10_ to air) and $m_{\mathrm{road}\;\mathrm{particles}}$ is the amount of road particles worn from the road by the tire studs (kg PM_10_ to air). PM_10_ is particulate matter of sizes < 10 μm. The endpoint characterization factor was obtained from the ReCiPe 2008 method, using the hierarchist perspective [30]. The hierarchist perspective was applied in order to be consistent with the impact assessment data applied in Section 2.3, and reflects common policy principles regarding, for example, time frames. The amount of particles worn from the road was calculated according to the following:

(8) $$m_{\mathrm{road}\;\mathrm{particles}}=E_{f}\times L_{\mathrm{veh}\;\mathrm{km}\;\mathrm{car}}\times L_{\mathrm{tire}},$$

where $E_{f}$ is an emission factor (kg PM_10_ to air/vehicle km) and $L_{\mathrm{veh}\;\mathrm{km}\;\mathrm{car}}$ is the average number of vehicle km that a studded passenger car drives per year (vehicle km/year). The emission factor for PM_10_ from road wear caused by tire studs was set to 20–50 mg PM_10_ to air/vehicle km, based on measurements in the two Swedish cities of Umeå and Gothenburg [31]. Swedish statistics were employed in order to obtain an average value for the number of vehicle km driven by a passenger car with studded tires during winter. The statistics were applied, under the assumption that cars with different types of winter tires (i.e., studded or non-studded ones) drive equally far during winter, according to the following:

(9) $$L_{\mathrm{veh}\;\mathrm{km}\;\mathrm{car}}=\frac{L_{\mathrm{veh}\;\mathrm{km}\;\mathrm{tot}\;\mathrm{car}}\times S_{\mathrm{veh}\;\mathrm{km}\;\mathrm{winter}}}{N_{\mathrm{tot}\;\mathrm{car}}},$$

where $L_{\mathrm{veh}\;\mathrm{km}\;\mathrm{tot}\;\mathrm{car}}$ is the total number of vehicle km driven by the $N_{\mathrm{tot}\;\mathrm{car}}$ (vehicle km/year) and $S_{\mathrm{veh}\;\mathrm{km}\;\mathrm{winter}}$ is the share of these vehicle kilometers that were driven during winter (–). In 2013–2017, the total of 4,500,000–4,800,000 passenger cars in Sweden [32] drove 63,000–68,000 million vehicle km [33]. According to Öberg [34], approximately 35% of the annual vehicle km occur during winter. The same lifetime of studded tires was applied as in Section 2.1.

### 2.3. System Boundary 2: Production System Emissions

The contribution of production system emissions to human health impacts was calculated by applying the ReCiPe 2008 method with the hierarchist perspective [30]. The following six different midpoint impact categories contribute to human health impacts: climate change (kg CO_2_ eq to air), ozone depletion (kg CFC-11 eq to air), human toxicity (kg 1,4-DCB eq to urban air), photochemical oxidant formation (kg NMVOC eq to urban air), particulate matter formation (kg PM_10_ to air), and ionizing radiation (kg U_235_ eq to air). The total human health impact from production system emissions was thus calculated according to the following:

(10) $${\mathrm{DALY}}_{\mathrm{prod}\;\mathrm{em}}=\sum_{i}{\;\mathrm{CF}}_{i}\times I_{i},$$

where ${\mathrm{CF}}_{i}$ is the endpoint characterization factor for midpoint impact category *i* (e.g., DALY/kg CO_2_ eq to air) and $I_{i}$ is the life cycle contribution to *i* (e.g., kg CO_2_ eq to air). China dominates the global tungsten production [22]. However, China’s share of the global WC-Co production is less than half, and only 23% of this was exported in 2015 [35,36]. The life cycle inventory (LCI) data from Furberg et al. [37] for the typical non-Chinese WC-Co production was therefore used to calculate life cycle impact assessment (LCIA) data for the production system of tire stud pins using ReCiPe 2008 [30] and the uniform system for the evaluation of substances adapted for LCA purposes (USES-LCA) [38]. Furthermore, the production system emissions related to the aluminum bodies in one passenger car’s tire studs were calculated based on the weight of the aluminum body (0.85 g [15]) and the LCIA data from the Ecoinvent database [39] for “aluminium production, primary, ingot, rest of the world (RoW)” (allocation by cut-off and ReCiPe 2008 with a hierarchist perspective).

### 2.4. System Boundary 2: Production System Accidents

Scanlon et al. [40] provide work environment characterization factors (WE-CFs) based on U.S. industrial safety and health data, as well as related quantities of industrial outputs. These WE-CFs can be used to calculate the occupational human health impacts of an industrial activity by multiplying physical data, represented by LCI data, with the corresponding WE-CF, according to the following:

(11) $${\mathrm{DALY}}_{\mathrm{prod}\;\mathrm{acc}}=\sum_{n}m_{n}\times {\mathrm{WE-CF}}_{n},$$

where $m_{n}$ is the physical data associated with industrial activity *n* (e.g., kg output) and ${\mathrm{WE-CF}}_{n}$ is the work environment characterization factor for *n* (e.g., DALY/kg output). The WE-CFs from the authors of [40] were considered to adequately represent the typical non-Chinese production of WC-Co [37] and aluminum production. Unfortunately, the lack of inclusion of these WE-CFs in the existing LCA databases makes it difficult to be as comprehensive regarding the production system accidents as for the production system emissions. The approach taken here was therefore to include the main parts of the life cycle from which the largest contribution is likely to occur. The LCI data was obtained from the literature [37] for the foreground system of WC-Co production, consisting of mining, transport, hydrometallurgy, pyrometallurgy, and powder metallurgy. Production accidents related to these main processes where calculated using Equation (11). In addition, for each of these five main processes of the WC-Co production, the contribution to occupational health impacts from their sub-processes, for example, diesel and cobalt production, were also included. Using the Ecoinvent database [39] for mapping the up- and down-stream life cycles, the main production routes for these sub-processes and their main inputs were also considered. The occupational accidents from the production of the aluminum bodies (see Section 2.3) were accounted for in the same way. A flowchart showing the included processes in the production system accident calculations is shown in Section S2 in the SM. There, a list of the considered main inputs and outputs, the corresponding Ecoinvent processes, and the corresponding industrial activities are also shown. The electricity and heat used in the background system were not accounted for, because of a lack of information about their specific geographical origin and thus what type of electricity production it is, which is required in order to assign the proper WE-CF to it [40].

The occupational health impacts from cobalt mining in the DRC were expected to be considerably higher than the U.S.-based data would imply, as this mining is mainly artisanal and workers are exposed to a high level of accidents [41,42]. Therefore, using the same approach as the authors of [40], a new WE-CF was derived for the sub-process of artisanal cobalt mining (${\mathrm{WE-CF}}_{\mathrm{Co}}$), according to the following:

(12) $${\mathrm{WE-CF}}_{\mathrm{Co}}=\frac{{\mathrm{DALY}}_{\mathrm{miner},\;\mathrm{fatal}\;\mathrm{acc}}+{\mathrm{DALY}}_{\mathrm{miner},\;\mathrm{acc}}}{m_{\mathrm{Co},\;\mathrm{DRC}}},$$

(13) $${\mathrm{DALY}}_{\mathrm{miner},\;\mathrm{fatal}\;\mathrm{acc}}=N_{\mathrm{miners}}\times S_{\mathrm{fatal}\;\mathrm{acc}}\times {(\mathrm{LEX}}_{\mathrm{DRC}}-L),$$

(14) $${\mathrm{DALY}}_{\mathrm{miner},\;\mathrm{acc}}=N_{\mathrm{miners}}\times N_{\mathrm{acc}\;\mathrm{per}\;\mathrm{person}}\times \underset{k}{\sum }S_{\mathrm{acc},\;k}\times {\mathrm{DW}}_{k}\times L_{k},$$

where ${\mathrm{DALY}}_{\mathrm{miner},\;\mathrm{fatal}\;\mathrm{acc}}$ is the number of DALY lost annually in fatal accidents in the DRC due to cobalt mining (year), ${\mathrm{DALY}}_{\mathrm{miner},\;\mathrm{acc}}$ is the number of DALY lost annually in non-fatal accidents in the DRC due to cobalt mining (year), $m_{\mathrm{Co},\;\mathrm{DRC}}$ is the amount of cobalt mined in the DRC per year (kg/year), $N_{\mathrm{miners}}$ is the numbers of miners involved in cobalt mining in the DRC (–), $S_{\mathrm{fatal}\;\mathrm{acc}}$ is the share of fatal accidents of miners involved (–), ${\mathrm{LEX}}_{\mathrm{DRC}}$ is the life expectancy in the DRC (year), *L* is the age at death (year), $N_{\mathrm{acc}\;\mathrm{per}\;\mathrm{person}}$ is the number of accidents per miner per year (accidents/year), $S_{\mathrm{acc},\;k}$ is the share of mining accidents that are of injury type *k*, ${\mathrm{DW}}_{k}$ is the disability weight for injury type *k*, and $L_{k}$ is the time that a person spends with an injury of type *k* until recovery or death. In 2011–2015, 52,000–63,000 metric tonnes of cobalt were mined per year in the DRC [21]. About 250,000 people are involved in heterogenite mining, which is the most abundant cobalt mineral in Katanga, DRC, where around half of the world’s mineable cobalt reserves are located [41,43]. Fatal accidents in general artisanal mining occur to 2.5% of the miners [44], and this was used as an estimate for the fatal accidents in artisanal cobalt mining, specifically. The life expectancy in the DRC was 58–60 years in 2012–2016 [29]. Statistics on the number of non-fatal accidents per miner and common accident types were provided from a survey among the artisanal miners in Katanga, conducted in 2009 [42]. On average, 2.2 accidents per miner, causing injuries such as bruises, wounds, and fractures, were reported, and 90% of the miners where in between the ages of 19–37 years. The age of these miners was used as the age at death in the calculations of ${\mathrm{DALY}}_{\mathrm{miner},\;\mathrm{fatal}\;\mathrm{acc}}$. The two most common injuries reported that cause disability, following the literature [27], were wounds and fractures, representing 44% and 5.4% of the reported injuries, respectively. Thus, *k* = {fracture, wound} in Equation (14). Disability weights for wounds and fractures are 0.006 and 0.01–0.4, respectively [27], and the time spent with a disability until recovery is one to six months for fractures [28], and is assumed to be one to four weeks for wounds.

### 2.5. System Boundary 3: Conflict Health Impacts

The number of years lost due to revenues from the mineral cobalt was calculated according to the following:

(15) $${\mathrm{DALY}}_{\mathrm{conflict}}=m_{\mathrm{Co}}\times {\mathrm{CF}}_{\mathrm{conflict},\;\mathrm{Co}},$$

where $m_{\mathrm{Co}}$ is the mass of cobalt mined in the DRC for one studded passenger car (kg) and ${\mathrm{CF}}_{\mathrm{conflict},\;\mathrm{Co}}$ is the number of years lost per cobalt mined—basically a characterization factor for the conflict-related health impacts of the material (year/kg). The mass of cobalt mined was calculated based on a 6–10% cobalt content of the 0.10–0.22 kg WC-Co pins, then, following the Ecoinvent process ‘cobalt production (GLO)’ until its extraction, in order to get a figure for the required amount of mined cobalt [39]. Values for ${\mathrm{CF}}_{\mathrm{conflict},\;\mathrm{Co}}$ were obtained from the inclusive scenario of Furberg et al. [45], who calculated the DALY lost for a conflict mineral *i* and time period *j* according to the following:

(16) $${\mathrm{CF}}_{\mathrm{conflict},\;\mathrm{Co},\;j}=\frac{N_{j}\times {(\mathrm{LEX}}_{j}{-L}_{j})\times P_{\mathrm{Co},\;j}}{\sum_{i,\;j}P_{i,\;j}\times m_{i,\;j}},$$

where *N* is the number of premature direct deaths in the DRC due to the conflict (–), *LEX* is the national life expectancy in the DRC (year), *L* is the average age at death (year), *P* is the average global market price (USD/ton), *P_Co_* is the average global market price for cobalt (USD/ton) and *m* is the production in the DRC (ton). In Equation 14, *i* = {tin, tantalum, tungsten, gold, copper, cobalt, diamond}. ${\mathrm{CF}}_{\mathrm{conflict},\;\mathrm{Co}}$ was set to 1.1 × 10^−4^ in the LS and 3.2 × 10^−4^ in the HS [45]. These values were obtained by applying a range of 490–2200 deaths for *N* [46] and 57–59 years for *LEX* [29]. The age at death was 2.5 years for 46% of the deaths and 10–30 years for 54% of the deaths [10]. Data applied for *P* and *m* is presented in Section S3 in the SM. A more detailed description of the derivation of ${\mathrm{CF}}_{\mathrm{conflict},\;\mathrm{Co}}$ can be found in ref. [45].

## 3. Results

**Figure 2:**
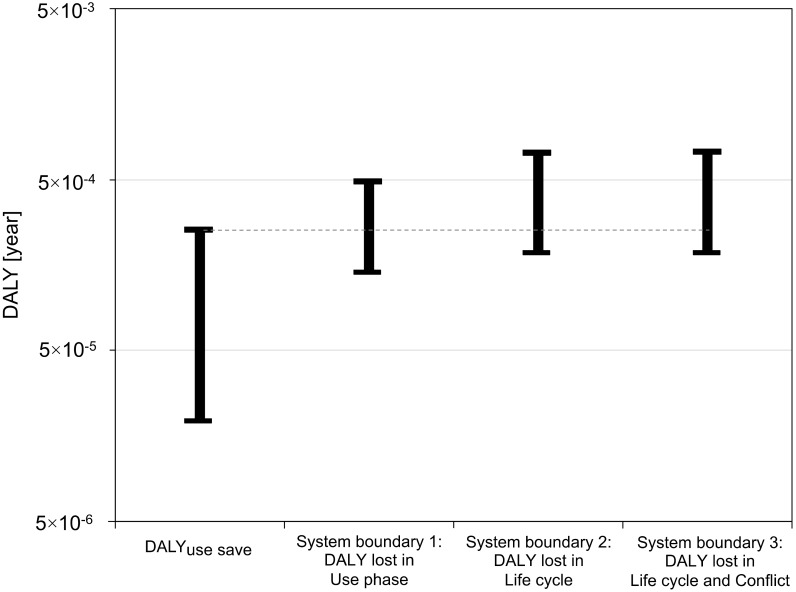
Results per tire studs in a studded passenger car used in Scandinavia, for the three system boundaries. ${\mathrm{DALY}}_{\mathrm{use}\;\mathrm{save}}$ = years saved by using studded tires instead of non-studded winter tires during winter.

The results in DALY per tire studs used in a Scandinavian studded passenger car are presented in Figure 2. They show that the use of tire studs results generally in more DALY lost than saved. The two scenarios (LS and HS) illustrated by the upper and lower intervals shows that there is an overlap between the HS for DALY use saved and the LS for DALY lost. This overlap is quite small and decreases when expanding from system boundary 1 to system boundary 3. The most optimistic estimate for the benefits is somewhat larger than the very optimistic estimate for the damages. The positive health impacts of the tire studs are thus, at the very best, somewhat larger than the lowest estimate for the negative health impacts.

At system boundary 1, the years saved from using studded tires is lower than the years lost from the use phase emissions in both the LS and the HS. However, there is an overlap, such that the HS of the use phase saved is slightly higher than the LS of the use phase emissions. The system boundary 2, expanding from the use phase to the life cycle perspective of the tire studs by including the additional factors of production system emissions and accidents, shows a smaller overlap between the years saved and the years lost, compared to system boundary 1. The largest contribution to the years lost from production system emissions comes from the production of the aluminum bodies (91%) in the LS, while the largest contribution comes from the production of the WC-Co tire stud pins (69%) in the HS, specifically, the treatment of separated slag materials in tungsten mining. The largest contribution to the years lost due to production system accidents originate from the artisanal cobalt mining (80–90%), which is hardly surprising considering the harsh conditions of the miners [42]. Considering system boundary 3, the years lost due to conflicts arising from the revenues of the cobalt mining are at least one order of magnitude smaller than the number of DALY saved from the use of studded tires. Overall, 67–77% of the negative human health impacts occurs at system boundary 1, while the additional factors included in system boundary 2 (i.e., ${\mathrm{DALY}}_{\mathrm{prod}\;\mathrm{em}}$ and ${\mathrm{DALY}}_{\mathrm{prod}\;\mathrm{acc}}$) and system boundary 3 (i.e., ${\mathrm{DALY}}_{\mathrm{conflict}}$) cover 23–31% and 0.4–1.2%, respectively. Although the use phase thus accounts for most of the negative health impact caused for the tire studs, in particular the production system emissions and occupational accidents in the production system also contribute significantly. The exact magnitudes of the different health impacts (*DALY_use save_*, *DALY_use em_*, *DALY_prod em_*, *DALY_prod acc_* and *DALY_conflict_*) and the result for *DALY_tire stud_* are presented in Section S4 in the SM.

In addition to the DALY caused generally being higher than the DALY saved for tire studs, there is also a partly uneven distribution of the health impacts over the life cycle. Mostly, about the same amount of Scandinavian people that benefitted from the DALY saved are also affected by the DALY caused, assuming then that car driving is well spread over the general population. However, 23–33% of the negative health impacts occur outside of Scandinavia, mainly due to production system emissions and accidents. This calls into question the use of tire studs not only regarding the in general negative net health impacts, but also from a justice perspective, regarding which people are benefited versus impacted.

## 4. Discussion

There are a number of additional contributions to human health that could be subject to future research in order to improve the analysis conducted in this study. However, as will be discussed below, the inclusion of these additional contributions would probably not change the main conclusion that tire studs in general do not provide net health benefits from a life cycle perspective (i.e., their inclusion would not alter the net health balance). Their inclusion would probably rather increase the negative human health impacts and thereby strengthen the conclusion.

Regarding contributions to positive health impacts, Feschet et al. [47] provided a method for the inclusion of human health improvements from increased income, called the Preston pathway. However, that method is only justified for the parts of the life cycle located in poor countries and in countries where the generated wealth is spread over the countries’ economic sectors (i.e., countries with low corruption). These conditions were not expected to be fulfilled in this study, as most countries in the production system are not poor enough and wealth is generally not spread enough in the DRC, indicated by its 161 rank out of 180 countries, according to the corruption perception index in 2017 [48].

Regarding the additional contributions to negative health impacts, the noise caused by studded tires is generally higher than for other tires [49]. Methods for including human health impacts from noise in LCA have been suggested, for example, by Ongel [50]. However, such methods are generally dependent on specific input data, such as traffic volume and speed, which might be obtained from on-site measurements, but were not available for the more generic assessment conducted in this study. Furthermore, for the assessment of life cycle occupational accidents, the authors of [40] concluded that infrastructure-related processes such as the construction of capital goods, which are often excluded in LCA studies and also in this study, often have a large contribution to the total human health impacts (72–96% in their case studies). The occupational accidents calculated in this study for the cobalt mining, specifically, are also probably underestimated. Reports on accidents in artisanal mining are generally very limited [41]. According to the literature [42] used in this study, their survey is influenced by the ‘healthy worker effect’, meaning that typically, only healthy workers are present to answer questionnaires at the time of investigation. Thus, fatal accidents or accidents leading to permanent disability are not well captured and the results thus do not reflect the actual severity of accidents. The assessment of lives lost due to conflict is also subject to considerable uncertainties. One main issue is that the calculation in this study is limited to direct deaths. Armed conflict also indirectly affects health, for example, by disrupting health-care services and damaging public health infrastructure, and the share of excess indirect deaths in historical African conflicts might have been as high as 80–95% [51,52].

The results from this study raise the question as to whether there are any potential alternatives to tire studs that can achieve the same function. Electronic stability control (ESC), which is already used to a large extent in passenger cars, is one such alternative that has been posed [53]. Elvik [53] found that if all Norwegian cars have ESCs, the use of studded tires can be reduced but not completely eliminated if an increase in accident rates is to be avoided. Solutions to problems associated with the use of studded tires have also been suggested and include the development of wear-resistant asphalt for the abatement of high costs associated with damaged roads [54]. However, this type of measure could potentially also bring additional negative health impacts (e.g., during asphalt production). We therefore recommend studies of the net health impacts of different alternatives to tire studs.

## 5. Conclusions

This study shows that the purpose of tire studs to reduce health impacts is, in general, not justified, nor when only the use phase is considered nor from a broader life cycle perspective. The health impacts saved by a Scandinavian studded passenger car were shown to be generally outweighed by different negative human health impacts. These were dominated by use phase particle emissions (67–77%), while negative human health impacts from occupational accidents during artisanal cobalt mining also contributed significantly (8–18%). The latter contribution also causes health impacts far away from the place where the studs are used to provide health benefits. In total, 23–33% of the negative human health impacts occur outside Scandinavia. By taking a broader perspective, these results inform and widen the current debate that so far has been limited to the use phase of studded tires. Assessments of alternatives, by applying a life cycle perspective, to studded tires are recommended.

## Supplementary Materials

The following are available online at http://www.mdpi.com/1660-4601/15/8/1774/s1, Table S1: Description of parameters. Table S2: Input data for calculations applied in the low and high impact scenarios, Figure S1: Flowchart for the included foreground and background processes in the production system of tire studs, Table S3: Ecoinvent processes and industrial categories applied for inputs and outputs in the foreground and background system, Table S4: Prices of minerals in USD/ton, Table S5: Amounts of conflict minerals mined in the Democratic Republic of the Congo, Table S6: Contributions to health impacts.
