# Supplementary material for: Live and Let Die? Life Cycle Human Health Impacts from the Use of Tire Studs
Source: Int J Environ Res Public Health. 2018 Aug 17;15(8):1774. doi: 10.3390/ijerph15081774 (PMC6121463; doi:10.3390/ijerph15081774)
Supplement: Supplementary file 1 [file ijerph-15-01774-s001.pdf]

Supplementary materials for

Live and Let Die? Life Cycle Human Health Impacts from the Use of Tire Studs

Anna Furberg*, Rickard Arvidsson, Sverker Molander
Division of Environmental Systems Analysis, Chalmers University of Technology, Vera Sandbergs Allé 8, SE 412 96 Gothenburg, Sweden

* Corresponding author. E-mail: anna.furberg@chalmers.se

Table of Contents

1. Input Data for Calculations......1
2. Data for Production System Accidents......11
3. Prices and Amounts of Conflict Minerals......17
4. Resulting Contributions to Health Impacts......18
5. References......19

1. Input Data for Calculations

Table S1. Description of parameters. DALY=disability-adjusted life years and DRC=the Democratic Republic of the Congo.

**Parameter [unit]**	**Description**
**System boundary 1: Lives saved in the use phase ($DALY_{use\ save}$)**	
R [-]	Expected accident reduction rate if studded tires are used instead of non-studded winter tires
N_{acc} [accidents/year]	Number of accidents that an average passenger car with non-studded winter tires is involved in during winter
$N_{acc\ tot}$ [-]	Total number of accidents with passenger cars using non-studded winter tires during winter
$N_{tot\ car}$ [-]	Total number of registered passenger cars
$S_{car\ non\text{-}studded}$ [-]	Share of passenger cars with non-studded winter tires during winter
L_{tire} [year]	Lifetime of studded tires
$DALY_{car\ acc}$ [year/accident]	Number of DALY per passenger car accident
$N_{fatal\ car\ acc}$ [-]	Annual number of persons who lost their lives in fatal passenger car accidents
LEX_{Sca} [year]	Life expectancy in Scandinavia
L_{death} [year]	Age of the person at death in a fatal passenger car accident
$N_{severe\ car\ acc}$ [-]	Annual number of persons who got severely injured in passenger car accidents
DW_{severe} [-]	Disability weight for a severe injury
L_{severe} [year]	Length of disability for a severe injury
$N_{slight\ car\ acc}$ [-]	Annual number of persons who got slightly injured in passenger car accidents
DW_{slight} [-]	Disability weight for a slight injury
L_{slight} [year]	Length of disability for a slight injury
$N_{tot\ car\ acc}$ [-]	Annual total number of passenger car accidents (i.e. fatal, severe and slight passenger car accidents)

Parameter [unit]	Description
**System boundary 1: Particle emissions in the use phase ($DALY_{use\ em}$)**	
$CF_{PM10 \rightarrow DALY}$ [year/kg PM_{10} to air]	Endpoint characterization factor converting emissions of PM_{10} to DALY
$m_{road\ particles}$ [kg PM_{10} to air]	Amount of road particles worn from the road by the tire studs in a studded passenger car
E_f [kg PM_{10} to air /vehicle km]	Emission factor of PM_{10} from a studded passenger car
$L_{veh\ km\ car}$ [vehicle km/year]	Average number of vehicle km that a studded passenger car drives per year
$L_{veh\ km\ tot\ car}$ [vehicle km/year]	Total number of vehicle km driven by the total number of registered passenger cars
$S_{veh\ km\ winter}$ [-]	Share of the $L_{veh\ km\ tot\ car}$ that were driven during winter
$N_{tot\ car}$ [-]	Total number of registered passenger cars
L_{tire} [year]	Lifetime of studded tires
**System boundary 2: Production system emissions ($DALY_{prod\ em}$)**	
CF_i [e.g. DALY/kg CO_2 eq]	Endpoint characterization factor for midpoint impact category *i*
I_i [e.g. kg CO_2 eq]	Life cycle contribution to midpoint impact category *i*
**System boundary 2: Production system accidents ($DALY_{prod\ acc}$)**	
m_n [e.g. kg output]	Physical data associated with industrial activity *n*
$WE\text{-}CF_n$ [e.g. DALY/kg output]	Work environment characterization factor for industrial activity *n*
$WE\text{-}CF_{Co}$ [DALY/kg]	Work environment characterization factor for artisanal cobalt mining
$DALY_{miner\ fatal\ acc}$ [year]	Number of DALY lost annually in fatal accidents in the DRC due to cobalt mining
N_{miners} [-]	Numbers of miners involved in cobalt mining in the DRC
$S_{fatal\ acc}$ [-]	Share of miners involved in cobalt mining in the DRC that are subject to fatal accidents
LEX_{DRC} [year]	Life expectancy in the DRC
L [year]	Age of miners at death caused by a fatal accident
$DALY_{miner\ acc}$ [year]	Number of DALY lost annually in non-fatal accidents in the DRC due to cobalt mining
$N_{acc\ per\ person}$ [-]	Number of accidents per miner per year

Parameter [unit]	Description
$S_{acc, fracture}$ [-]	Share of mining accidents that are of injury type; fracture
$DW_{fracture}$ [-]	Disability weight for injury type; fracture
$L_{fracture}$ [year]	The time that a person spends with an injury of type; fracture, until recovery or death
$S_{acc, wound}$ [-]	Share of mining accidents that are of injury type; wound
DW_{wound} [-]	Disability weight for injury type; wound
L_{wound} [year]	The time that a person spends with an injury of type; wound, until recovery or death
$m_{Co\ DRC}$ [kg]	Amount of cobalt mined in the DRC per year
**System boundary 3: Conflict ($DALY_{conflict}$)**	
m_{Co} [kg]	Mass of cobalt mined in the DRC for one studded passenger car
$CF_{conflict, Co}$ [year/kg]	Number of years lost per cobalt mined
N_j [-]	Number of premature direct deaths in the DRC due to the conflict in time period *j*
LEX_j [year]	National life expectancy in the DRC in time period *j*
L_j [year]	Average age at death in time period *j*
$P_{i,j}$ [USD/ton]	Average global market price for conflict mineral *i* in time period *j*
$m_{i,j}$ [kg]	Production in the DRC of conflict mineral *i* in time period *j*

Table S2. Input data for calculations applied in the low and high impact scenarios. The low and high impact scenarios are denoted LS and HS, respectively. Descriptions of the parameters are provided in Section 2.1 to 2.5 in the article. WC-Co= tungsten carbide with cobalt, Al=aluminum and DRC=the Democratic Republic of the Congo.

Parameter [unit]	Value		Comment	Reference
	LS	HS		
**Functional unit (the weight of the tire studs in four studded tires)**				
Functional unit [kg]	0.1 (WC-Co)<br>0.4 (Al)	0.22 (WC-Co)<br>0.46 (Al)		Calculated
Weight of a tire stud WC-Co pin [g]	0.2	0.4		Furberg, et al. [1]
Weight of a tire stud Al body [g]	0.85	0.85		Peltola and Wikström [2]
Number of tire studs in four studded tires [-]	480	540	The number of tire studs in one studded tire is 119-135.	Ref. [1]
Cobalt content in a WC-Co pin [%]	6	10		Ref. [1]
**System boundary 1: Lives saved in the use phase ($DALY_{use\ save}$)**				
R [-]	0.02	0.05	Compared to non-studded winter tires, studded ones reduce accident rates with 2% on bare roads and 5% on icy or snowy roads.	Elvik [3]
N_{acc} [accidents/year]	0.00051	0.0011		Calculated
$N_{acc\ tot}$ [-]	860	1200	The lowest and highest annual number of accidents with passenger cars using non-studded winter tires in Norway in winter (November to April) during the time period of 2012/2013-2016/2017 were 860 in the winter of 2016/2017 and 1200 in the winter of 2012/2013, respectively.	SSB [4]
$N_{tot\ car}$ [-]	2 700 000	2 400 000	The lowest and highest annual number of total registered passenger cars in Norway during the time period of 2012-2016 were 2 400 000 in 2012 and 2 700 000 in 2016, respectively.	SSB [5]

Parameter [unit]	Value		Comment	Reference
	LS	HS		
$S_{car\ non\text{-}studded}$ [-]	0.64	0.46	The lowest and highest annual average share of passenger cars with non-studded winter tires in Norway in the time period of 2012-2016 were 46% in 2014 and 64% in 2016, respectively.	NPRA [6]
L_{tire} [year]	6	7	Winter tires are approximately used for 6-7 years.	Swedish Transport Administration [7]
$DALY_{car\ acc}$ [year/accident]	0.32	0.65		Calculated
$N_{fatal\ car\ acc}$ [-]	120	140	The lowest and highest annual number of persons who lost their lives in fatal passenger car accidents in Sweden during the time period of 2013-2017 were 120 in 2014 and 140 in 2015, respectively. See information on "Persons killed, severely and slightly injured in road traffic accidents reported by the police by age, group of road users and sex" from the reference.	Transport Analysis [8]
LEX_{Sca} [year]	81	82	The lowest and highest average life expectancy in Scandinavia (i.e. Sweden, Norway and Denmark) during the time period of 2012-2016 were 81 in 2012 and 82 in 2016, respectively.	World Bank [9]
L_{death} [year]	Many different	Many different	This data was given for each individual fatal accident as described in the statistics from the reference. When ranges were given for the age of the affected person (e.g. 35-44 year), the high value (e.g. 44 year) was applied in the LS and the low value (e.g. 35 year) in the HS. For the age group of 75+, L_{death} was set equal to LEX_{Sca} in the LS. For exact values, see the reference.	Transport Analysis [8]
$N_{severe\ car\ acc}$ [-]	1 300	1 600	The lowest and highest annual number of persons who got severely injured in passenger car accidents in Sweden during the time period of 2013-2017 were 1 300 in 2017 and 1 600 in 2013, respectively. See information on "Persons killed, severely and slightly injured in road traffic accidents reported by the police by age, group of road users and sex" from the reference.	Transport Analysis [8]

Parameter [unit]	Value		Comment	Reference
	LS	HS		
DW_{severe} [-]	0.01	0.4	The average disability weight of a fracture in the hand, short term, with or without treatment at 0.01 and of a fracture in the neck of femur, long term, without treatment at 0.4 were assumed in the LS and HS, respectively.	Salomon, et al. [10]
L_{severe} [year]	0.083	0.5	Time of recovery from fractures in general is about 1-6 months.	MEDIBAS [11]
$N_{slight\ car\ acc}$ [-]	10 000	12 000	The lowest and highest annual number of persons who got slightly injured in passenger car accidents in Sweden during the time period of 2013-2017 were 10 000 in 2014 and 12 000 in 2013, respectively. See information on "Persons killed, severely and slightly injured in road traffic accidents reported by the police by age, group of road users and sex" from the reference.	Transport Analysis [8]
DW_{slight} [-]	0.008	0.008	The average disability weight of other injuries of muscles and tendons (consisting of sprains, strains and dislocations other than shoulder, knee or hip) at 0.008 was assumed for both the LS and HS.	Ref. [10]
L_{slight} [year]	0.042	0.5	Time of recovery from a sprained ankle is about two weeks to half a year which was assumed to be valid for sprains in general.	MEDIBAS [11]
$N_{tot\ car\ acc}$ [-]	10 000	9 100	The total number of passenger car accidents were obtained by summing the number of such accidents causing fatal or severe personal injury with the number of slightly injured passenger car drivers (the latter was used as an assumption for the total number of passenger car accidents with slight personal injury due to limited data). See information on "Road traffic accidents with fatal or severe personal injury reported by the police including persons killed or severely injured, by involved type of traffic elements" and "Persons killed, severely and slightly injured in road traffic accidents reported by the police by age, group of road users and sex" from the reference.	Transport Analysis [8]

Parameter [unit]	Value		Comment	Reference
	LS	HS		
**System boundary 1: Particle emissions in the use phase ($DALY_{use\ em}$)**				
$CF_{PM10 \rightarrow DALY}$ [year/kg PM_{10} to air]	$2.6 \cdot 10^{-4}$	$2.6 \cdot 10^{-4}$	Hierarchist perspective.	Goedkoop, et al. [12]
$m_{road\ particles}$ [kg PM_{10} to air]	0.55	1.9		Calculated
E_f [kg PM_{10} to air /vehicle km]	$20 \cdot 10^{-6}$	$50 \cdot 10^{-6}$	Data from measurements conducted in the cities of Umeå and Gothenburg in Sweden.	Ferm and Sjöberg [13]
$L_{veh\ km\ car}$ [vehicle km/year]	4 600	5 300		Calculated
$L_{veh\ km\ tot\ car}$ [vehicle km/year]	$63\ 000 \cdot 10^{6}$	$68\ 000 \cdot 10^{6}$	The lowest and highest number of million vehicle km driven by all the passenger cars in Sweden in the time period of 2013-2017 was 63 000 in 2013 and 68 000 in 2017, respectively.	Transport Analysis [14]
$S_{veh\ km\ winter}$ [-]	0.35	0.35	The approximative share of the total vehicle km during a year that takes place during winter is 35%.	Öberg [15]
$N_{tot\ car}$ [-]	4 800 000	4 500 000	The lowest and highest number of total registered passenger cars in Sweden in the time period of 2013-2017 was 4 500 000 in 2013 and 4 800 000 in 2017, respectively.	Transport Analysis [16]
L_{tire} [year]	6	7	Winter tires are approximately used for 6-7 years.	Swedish Transport Administration [7]

Parameter [unit]	Value		Comment	Reference
	LS	HS		
**System boundary 2: Production system emissions ($DALY_{prod\ em}$)**				
CF_i [e.g. DALY/kg CO_2 eq to air]	Many different	Many different	Hierarchist perspective. *i* = climate change, ozone depletion, human toxicity, photochemical oxidant formation, particulate matter formation and ionizing radiation.	Ref. [12]
I_i [e.g. kg CO_2 eq to air]	Many different	Many different	LCIA data for typical non-Chinese WC-Co production was provided from ref. [17] and LCIA data for aluminum production (tire stud bodies) was obtained from ref. [18] applying allocation by cut-off, the ReCiPe 2008 method and the hierarchist perspective for "aluminium production, primary, ingot, RoW". The weight of a tire stud body is approximately 0.85g [2]. *i* = climate change, ozone depletion, human toxicity, photochemical oxidant formation, particulate matter formation and ionizing radiation.	Furberg, Arvidsson and Molander [17], Ecoinvent database [18], Peltola and Wikström [2]
**System boundary 2: Production system accidents ($DALY_{prod\ acc}$)**				
m_n [e.g. kg output]	Many different	Many different	LCI data for typical non-Chinese WC-Co production was provided from ref. [17] and LCIA data for aluminum production (tire stud bodies) was obtained from ref. [18] applying allocation by cut-off, the ReCiPe 2008 method and the hierarchist perspective for "aluminium production, primary, ingot, RoW". The weight of a tire stud body is approximately 0.85g [2]. See further Section 2.	Furberg, Arvidsson and Molander [17], Ecoinvent database [18], Peltola and Wikström [2]
$WE\text{-}CF_n$ [e.g. DALY/kg output]	Many different	Many different	Work environment characterization factors, based on United States safety and health data and related amounts of industrial outputs, were considered to adequately represent typical non-Chinese WC-Co production (except for cobalt mining) and aluminum production. See further Section 2.	Scanlon, et al. [19]
$WE\text{-}CF_{Co}$ [DALY/kg]	$2.1 \cdot 10^{-3}$	$5.0 \cdot 10^{-3}$		Calculated
$DALY_{miner\ fatal\ acc}$ [year]	130 000	250 000		Calculated

Parameter [unit]	Value		Comment	Reference
	LS	HS		
N_{miners} [-]	250 000	250 000	People involved in heterogenite mining [20], which is the most abundant cobalt mineral in Katanga in the DRC where about 50% of the global cobalt production takes place [21].	Elgstrand and Vingård [20], Decrée, Pourret and Baele [21]
$S_{fatal\ acc}$ [-]	0.025	0.025	In general, fatal accidents in artisanal mining occur to 2.5% of the miners involved in a year. This was assumed to be valid for artisanal cobalt mining specifically.	ILO [22]
LEX_{DRC} [year]	58	60	The lowest and highest average life expectancy in the DRC during the time period of 2012-2016 were 58 in 2012 and 60 in 2016, respectively.	World Bank [9]
L [year]	37	19	90% of the artisanal miners in the survey by the reference were 19-37 years and the age of these miners was assumed to be valid for the age at death of artisanal cobalt miners.	Elenge, et al. [23]
$DALY_{miner\ acc}$ [year]	55	6000		Calculated
$N_{acc\ per\ person}$ [-]	2.2	2.2	Average annual number of accidents per artisanal miner in Katanga.	Ref. [23]
$S_{acc,\ fracture}$ [-]	0.054	0.054	The survey by the reference reported 5.4% of injuries to be fractures.	Ref. [23]
$DW_{fracture}$ [-]	0.01	0.4	The average disability weight of a fracture in the hand, short term, with or without treatment at 0.01 and of a fracture in the neck of femur, long term, without treatment at 0.4 were assumed in the LS and HS, respectively.	Ref. [10]
$L_{fracture}$ [year]	0.083	0.5	Time of recovery from fractures in general is about 1-6 months.	MEDIBAS [11]
$S_{acc,\ wound}$ [-]	0.44	0.44	The survey by the reference reported 44% of injuries to be wounds.	Ref. [23]
DW_{wound} [-]	0.006	0.006	The average disability weight of a wound, short term, with or without treatment at 0.006 was assumed for both the LS and HS.	Ref. [10]
L_{wound} [year]	0.021	0.083	Time of recovery from wounds was assumed to be 1-4 weeks.	Assumption by the authors
$m_{Co\ DRC}$ [kg]	63 000 000	52 000 000	The lowest and highest values for cobalt mined in the DRC in 2011-2015 were 52 000 000 kg in 2012 and 63 000 000 kg in 2015, respectively.	USGS [24]

Parameter [unit]	Value		Comment	Reference
	LS	HS		
**System boundary 3: Conflict ($DALY_{conflict}$)**				
m_{Co} [kg]	0.0068	0.026	Based on 6-10% cobalt content of the WC-Co pins [1]. Following the Ecoinvent process "cobalt production [GLO]" until its extraction to get a figure for the amount of mined cobalt [18].	Ref. [1], Ecoinvent database [18]
$CF_{conflict,\ Co}$ [year/kg]	$1.1 \cdot 10^{-4}$	$3.2 \cdot 10^{-4}$		Calculated based on Furberg, et al. [25]
N [-]	490	2 200	The lowest and highest values for the number of direct deaths in the DRC due to the conflict in 2010-2014 were 490 in 2011 and 2 200 in 2013, respectively.	UCDP [26]
LEX [year]	57	59	The lowest and highest average life expectancy in the DRC during the time period of 2010-2014 were 57 in 2010 and 59 in 2014, respectively.	World Bank [9]
L [year]	2.5 (46%)<br>30 (54%)	2.5 (46%)<br>10 (54%)	The age at death was 2.5 years for 46% of the deaths and 10-30 years for 54% of the deaths.	Parsmo [27]
$P_{i,j}$ [USD/ton]	Many different	Many different	The lowest and highest values for prices for minerals i = {tin, tantalum, tungsten, gold, copper, cobalt, diamond} and time period j of 2010-2014 are presented in Section 3.	USGS [28], KP [29]
$m_{i,j}$ [kg]	Many different	Many different	The lowest and highest values for amounts of minerals i = {tin, tantalum, tungsten, gold, copper, cobalt, diamond} produced in the DRC in time period j of 2010-2014 are presented in Section 3.	USGS [24], KP [29]

2. Data for Production System Accidents

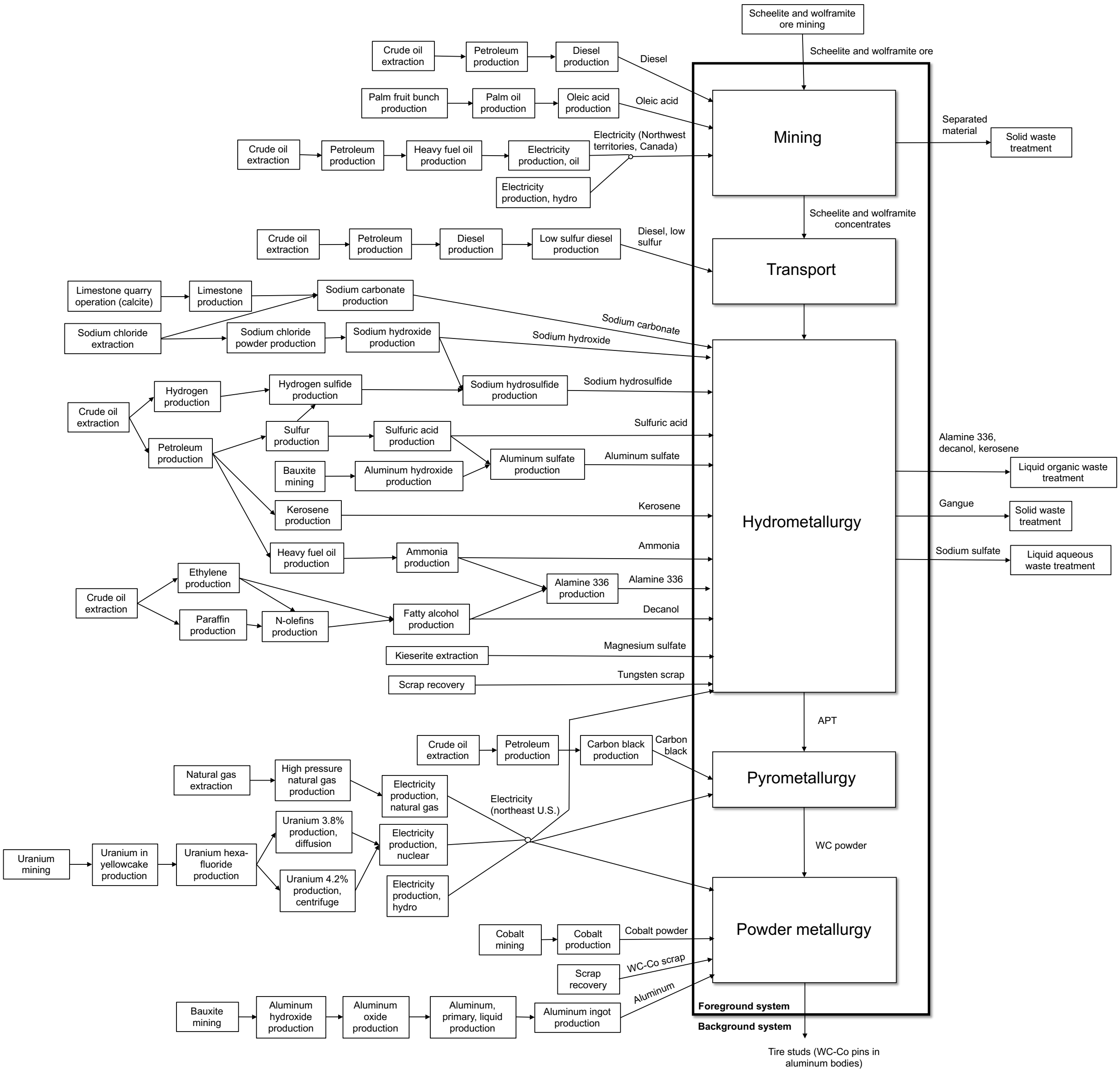

Figure S1. Flowchart for the included foreground and background processes in the production system of tire studs. APT=ammonium paratungstate, WC=tungsten carbide and WC-Co=tungsten carbide with cobalt.

Table S3. Ecoinvent processes and industrial categories applied for inputs and outputs in the foreground and background system. Note that no Ecoinvent process was available for Alamine 336, why a literature source was applied following ref. [17]. Furthermore, note that a specific calculation was conducted for the artisanal mining of cobalt, see Section 2.4 in the article. Some products are not given an industrial category but are still shown here since they were needed for further calculations.

Product	Ecoinvent process [18]	Industrial category [19]
Alamine 336	Alamine 336 production according to Vahidi and Zhao [30].	325199 All Other Basic Organic Chemical Manufacturing
Alamine 336 (liquid organic waste)	-	562211 Hazardous Waste Treatment and Disposal
Aluminum hydroxide	aluminium hydroxide production [GLO]	325188 All Other Basic Inorganic Chemical Manufacturing
Aluminum, ingot	aluminium, primary, ingot [kg]	331312 Primary Aluminum Production
Aluminum, primary, liquid	aluminium, primary, liquid [kg]	331312 Primary Aluminum Production
Aluminum oxide	aluminium oxide [kg]	325188 All Other Basic Inorganic Chemical Manufacturing
Aluminum sulfate	aluminium sulfate production, powder [RoW]	325188 All Other Basic Inorganic Chemical Manufacturing
Ammonia	ammonia production, partial oxidation, liquid [RoW]	325188 All Other Basic Inorganic Chemical Manufacturing
Ammonium paratungstate (APT)	-	325188 All Other Basic Inorganic Chemical Manufacturing
Bauxite	bauxite mine operation [GLO]	212299 All Other Metal Ore Mining
Calcite, in ground	-	212399 All Other Nonmetallic Mineral Mining
Carbon black	Carbon black production [RoW]	325182 Carbon Black Manufacturing
Cobalt	cobalt production [GLO]	331419 Primary Smelting and Refining of Nonferrous Metal (except Copper and Aluminum)
Cobalt, in ground	-	Special calculation conducted.
Crude oil	-	211111 Crude Petroleum and Natural Gas Extraction
Decanol	fatty alcohol production, petrochemical [RoW]	325199 All Other Basic Organic Chemical Manufacturing

**Product**	**Ecoinvent process [18]**	**Industrial category [19]**
Decanol (liquid organic waste)	-	562211 Hazardous Waste Treatment and Disposal
Diesel	petroleum refinery operation [RoW]	325110 Petrochemical Manufacturing
Diesel, low sulfur	diesel production, low-sulfur [RoW]	325110 Petrochemical Manufacturing
Electricity Canada, northwest territories	electricity, high voltage, production mix [CA-NT]	-
Electricity, hydro	-	221119 Other Electric Power Generation
Electricity, natural gas, combined cycle power plant	electricity production, natural gas, combined cycle power plant [NPCC, US only]	221112 Fossil Fuel Electric Power Generation
Electricity, natural gas, conventional power plant	electricity production, natural gas, conventional power plant [NPCC, US only]	221112 Fossil Fuel Electric Power Generation
Electricity nuclear, boiling water reactor	electricity production, nuclear, boiling water reactor [NPCC, US only]	221119 Other Electric Power Generation
Electricity nuclear, pressure water reactor	electricity production, nuclear, pressure water reactor [NPCC, US only]	221119 Other Electric Power Generation
Electricity, oil	electricity production, oil [CA-NT]	221112 Fossil Fuel Electric Power Generation
Electricity United States	electricity, high voltage, production mix, [NPCC, US only]	-
Ethylene	ethylene production, average [RoW]	325110 Petrochemical Manufacturing
Fatty alcohol	fatty alcohol production, petrochemical [RoW]	325199 All Other Basic Organic Chemical Manufacturing
Gangue (solid waste)	-	562212 Solid Waste Landfill
Heavy fuel oil	petroleum refinery operation [RoW]	325110 Petrochemical Manufacturing
Hydrogen	hydrogen cracking, APME [RoW]	325110 Petrochemical Manufacturing
Hydrogen sulfide	hydrogen sulfide production [RoW]	325188 All Other Basic Inorganic Chemical Manufacturing

Product	Ecoinvent process [18]	Industrial category [19]
Kerosene	petroleum refinery operation [RoW]	324110 Petroleum Refineries
Kerosene (liquid organic waste)	-	562211 Hazardous Waste Treatment and Disposal
Kieserite, 25% in crude or, in ground	-	212393 Other Chemical and Fertilizer Mineral Mining
Lime	limestone quarry operation [RoW]	327410 Lime Manufacturing
Magnesium sulfate	magnesium sulfate production [RoW]	325188 All Other Basic Inorganic Chemical Manufacturing
Natural gas, high pressure	petroleum and gas production, on-shore [RoW]	325120 Industrial Gas Manufacturing
Natural gas, in ground	-	211111 Crude Petroleum and Natural Gas Extraction
Nuclear fuel element for light water reactor	nuclear fuel element production, for pressure water reactor, UO2 4.2% & MOX [RoW]	-
N-olefins	n-olefins production [RoW]	325110 Petrochemical Manufacturing
Oleic acid	fatty acid production, from palm oil [RoW]	325199 All Other Basic Organic Chemical Manufacturing
Palm fruit bunch	palm fruit bunch production [RoW]	111339 Other Noncitrus Fruit Farming
Palm oil, crude	palm oil mill operation [RoW]	311223 Other Oilseed Processing
Paraffin	paraffin production [RoW]	324110 Petroleum Refineries
Petroleum	petroleum production, onshore [RoW]	324110 Petroleum Refineries
Scheelite ore	-	212299 All Other Metal Ore Mining
Scheelite and wolframite concentrates	-	212299 All Other Metal Ore Mining
Separated material	-	562212 Solid Waste Landfill
Sulfur	petroleum refinery operation [RoW]	324110 Petroleum Refineries
Sulfuric acid	sulfuric acid production [RoW]	325188 All Other Basic Inorganic Chemical Manufacturing
Sodium carbonate	soda production, solvay process [RoW]	325188 All Other Basic Inorganic Chemical Manufacturing

Product	Ecoinvent process [18]	Industrial category [19]
Sodium chloride	sodium chloride production, brine solution [RoW]	325181 Alkalies and Chlorine Manufacturing
Sodium chloride powder	sodium chloride production, powder [RoW]	325181 Alkalies and Chlorine Manufacturing
Sodium chloride, in ground	-	212393 Other Chemical and Fertilizer Mineral Mining
Sodium hydrosulfide	sodium hydrosulfide production [RoW]	325188 All Other Basic Inorganic Chemical Manufacturing
Sodium hydroxide	chlor-alkali electrolysis, membrane cell [RoW]	325181 Alkalies and Chlorine Manufacturing
Sodium sulfate (liquid aqueous waste)	-	221320 Sewage Treatment Facilities
Transport by lorry	transport, freight, lorry >32 metric ton, EURO3 [RoW]	484121 General Freight Trucking, Long-Distance, Truckload
Tungsten carbide (WC) powder	-	325188 All Other Basic Inorganic Chemical Manufacturing
Tungsten carbide with cobalt (WC-Co)	-	327910 Abrasive Product Manufacturing
Tungsten carbide with cobalt (WC-Co) scrap	-	562920 Materials Recovery Facilities
Tungsten scrap	-	562920 Materials Recovery Facilities
Uranium, enriched 3.8%	uranium production, diffusion, enriched 3.8% [RoW]	325188 All Other Basic Inorganic Chemical Manufacturing
Uranium, enriched 3.8%, in fuel element for light water reactor	uranium fuel element production, enriched 3.8%, for light water reactor [RoW]	-
Uranium enriched 4.2%, in fuel element	uranium fuel element production, enriched 4.2%, for light water reactor [RoW]	-
Uranium enriched 4.2%, per separative work unit	uranium production, centrifuge, enriched 4.2% [RoW]	-
Uranium hexaflouride	uranium hexafluoride production [RoW]	325188 All Other Basic Inorganic Chemical Manufacturing
Uranium, in yellowcake	uranium production, in yellowcake [RoW]	325188 All Other Basic Inorganic Chemical Manufacturing

Product	Ecoinvent process [18]	Industrial category [19]
Uranium ore, as U	uranium mine operation, underground [RoW]	212291 Uranium-Radium-Vanadium Ore Mining
Wolframite ore	-	212299 All Other Metal Ore Mining

3. Prices and Amounts of Conflict Minerals

Table S4. Prices of minerals in USD/ton. LS=low impact scenario, represented by the lowest value for the prices of minerals between 2010-2014, and HS=high impact scenario, represented by the highest value for the prices of minerals between 2010-2014.

Conflict mineral	LS	HS	Reference
Gold	40 000 000	54 000 000	USGS [28]
Tin	23 000	35 000	
Tantalum	150 000	340 000	
Tungsten	27 000	57 000	
Cupper	7 000	9 000	
Cobalt	28 000	40 000	
Diamond	44 000 000	43 000 000	KP [29]

Table S5. Amounts of conflict minerals mined in the Democratic Republic of the Congo. The unit is in ton except for diamond, which is given in carat. LS=low impact scenario, represented by the lowest value for the amount mined between 2010-2014, and HS=high impact scenario, represented by the highest value for the amount mined between 2010-2014.

Conflict mineral	LS	HS	Reference
Gold	12	31	USGS [24]
Tin	4 500	8 000	
Tantalum	250	520	
Tungsten	12	55	
Copper	420 000	1 000 000	
Cobalt	52 000	62 000	
Diamond	16 000 000	22 000 000	KP [29]

4. Resulting Contributions to Health Impacts

Table S6. Contributions to health impacts. The low and high impact scenarios are denoted LS and HS, respectively. DALY=disability-adjusted life years, $DALY_{tire\ stud}$=the net DALY of the tire studs in a studded Scandinavian passenger car, $DALY_{use\ save}$=years saved by using studded tires instead of non-studded winter tires during winter, $DALY_{total\ lost}$=years lost by using studded tires during winter, $DALY_{use\ em}$=years lost due to use phase emissions of road particles, $DALY_{prod\ em}$=years lost due emissions in the production system of tire studs, $DALY_{prod\ acc}$=years lost due to accidents in the production system of tire studs and $DALY_{conflict}$=years lost due to revenues from cobalt mineral mining. $DALY_{total\ lost}$ is the sum of $DALY_{use\ em}$, $DALY_{prod\ em}$, $DALY_{prod\ acc}$ and $DALY_{conflict}$. $DALY_{tire\ stud}$ is equal to $DALY_{use\ save}$ minus $DALY_{total\ lost}$.

Parameter [year]	LS	HS
$DALY_{tire\ stud}$	$-1.7 \cdot 10^{-4}$	$-4.7 \cdot 10^{-4}$
$DALY_{use\ save}$	$1.9 \cdot 10^{-5}$	$2.5 \cdot 10^{-4}$
$DALY_{total\ lost}$	$1.9 \cdot 10^{-4}$	$7.2 \cdot 10^{-4}$
$DALY_{use\ em}$	$1.4 \cdot 10^{-4}$	$4.8 \cdot 10^{-4}$
$DALY_{prod\ em}$	$2.4 \cdot 10^{-5}$	$8.0 \cdot 10^{-5}$
$DALY_{prod\ acc}$	$1.8 \cdot 10^{-5}$	$1.5 \cdot 10^{-4}$
$DALY_{conflict}$	$7.2 \cdot 10^{-7}$	$8.4 \cdot 10^{-6}$

5. References

1. Furberg, A.; Arvidsson, R.; Molander, S., Dissipation of Tungsten and Environmental Release of Nanoparticles from Tire Studs: A Swedish Case Study. *J Clean. Prod. (under review)* **2018**.
2. Peltola, P.; Wikström, E., Tyre stud derived tungsten carbide particles in urban street dust. *Boreal Environ. Res.* **2006,** 11, (3), 161-168.
3. Elvik, R., The effects on accidents of studded tires and laws banning their use: a meta-analysis of evaluation studies. *Accid. Anal. Prev.* **1999,** 31, (1), 125-134.
4. SSB (Statistisk sentralbyrå) (Eng. Statistics Norway) Road traffic accidents involving personal injury - 05247: Vehicles involved in road traffic accidents, by type of vehicle, tyre and accident. Latest update 20180416. Available online: https://www.ssb.no/en/statbank/table/05247/?rxid=5ad2fbe2-3a20-4d77-bcdd-c74350ad27c7 (accessed on 26 April 2018)
5. SSB (Statistisk sentralbyrå) (Eng. Statistics Norway) Registered vehicles - Registered vehicles by type of vehicle 1950-2017. Latest update 20180322. Available online: https://www.ssb.no/en/statbank/table/01960/?rxid=207552c2-f4a5-43b7-8232-1538d920f589 (accessed on 27 April 2018)
6. NPRA (Norwegian Public Roads Administration) Statens Vegvesen - Bruk av piggdekk 2017 (Eng. Norwegian Public Roads Administration - Use of studded tires 2017). Available online: https://www.vegvesen.no/fag/fokusomrader/trafikksikkerhet/Tilstandsundersokelser/_attachment/2167156?_ts=16193daab88&fast_title=Rapport+2017 (accessed on 27 April 2018)
7. Swedish Transport Administration *(Trafikverket) Undersökning av däcktyp i Sverige - vintern 2017 (januari - mars) (Eng. Investigation of tire type in Sweden - the winter of 2017 (January - March))*; 2017:184; Sweden: 2017.
8. Transport Analysis (Trafikanalys) Vägtrafikskador. (Eng. Road traffic accidents). Available online: https://www.trafa.se/vagtrafik/vagtrafikskador/ (accessed on 27 April 2018)
9. World Bank Life expectance at birth, total (years). Available online: https://data.worldbank.org/indicator/SP.DYN.LE00.IN (accessed on 27 April 2018)
10. Salomon, J. A.; Haagsma, J. A.; Davis, A.; de Noordhout, C. M.; Polinder, S.; Havelaar, A. H.; Cassini, A.; Devleesschauwer, B.; Kretzschmar, M.; Speybroeck, N.; Murray, C. J. L.; Vos, T., Disability weights for the Global Burden of Disease 2013 study. *Lancet Global Health* **2015,** 3, (11), e712-e723.
11. MEDIBAS Kunskapsstöd för hälso- och sjukvård (Eng. Knowledge support for health and medical service). Available online: https://medibas.se (accessed on 27 April 2018)
12. Goedkoop, M.; Heijungs, R.; Huijbregts, M.; De Schryver, A.; Struijs, J.; van Zelm, R. *ReCiPe 2008. A life cycle impact assessment method which comprises harmonised*

category indicators at the midpoint and endpoint level; Dutch Ministry of Housing, Spatial Planning and Environment (VROM): The Hague., 2013.
13. Ferm, M.; Sjöberg, K., Concentrations and emission factors for PM2.5 and PM10 from road traffic in Sweden. *Atmospheric Environ.* **2015,** 119, 211-219.
14. Transport Analysis *(Trafikanalys) Trafikarbete på svenska vägar (Eng. Vehicles kilometres on Swedish roads) Published 2018-04-27.*; 2018.
15. Öberg, G., Tema Vintermodell. Fordonskorrosion beroende på vintervägsaltning. Kunskapssammanställning. (Eng. Theme wintermodel. Vehicle corrosion dependent on salting on winter roads. Knowledge compilation) Swedish National Road and Transport Research Institute publication 2006:24. **2006**.
16. Transport Analysis (Trafikanalys) Fordon på väg. (Eng. Vehicles on road). Available online: https://www.trafa.se/vagtrafik/fordon/ (accessed on 27 April 2018)
17. Furberg, A.; Arvidsson, R.; Molander, S., Life Cycle Environmental Impacts of Cemented Carbide Production. *(manuscript in preparation)* **2018**.
18. Ecoinvent database, Version 3.4, 2017 In http://www.ecoinvent.org (accessed on 10th of November 2017). 2017.
19. Scanlon, K. A.; Lloyd, S. M.; Gray, G. M.; Francis, R. A.; LaPuma, P., An Approach to Integrating Occupational Safety and Health into Life Cycle Assessment: Development and Application of Work Environment Characterization Factors. *J. Ind. Ecol.* **2014,** 19, (1), 27-37.
20. Elgstrand, K.; Vingård, E., *Occupational Safety and Health in Mining*. Occupational and Environmental Medicine at Sahlgrenska Academy, University of Gothenburg: Gothenburg, 2013; Vol. 47.
21. Decrée, S.; Pourret, O.; Baele, J.-M., Rare earth element fractionation in heterogenite (CoOOH): implication for cobalt oxidized ore in the Katanga Copperbelt (Democratic Republic of Congo). *J. Geochem. Explor.* **2015,** 159, 290-301.
22. ILO, *(International Labour Organization) Social and labour issues in small-scale mines.* Geneva; OIT, 1999.
23. Elenge, M.; Leveque, A.; De Brouwer, C., Occupational accidents in artisanal mining in Katanga, D.R.C. *Int. J. Occup. Med. Environ. Health* **2013,** 26, (2), 265-274.
24. USGS *(U.S. Geological Survey) 2015 Minerals Yearbook Cobalt [Advanced release]. September, 2017.*; 2017.
25. Furberg, A.; Arvidsson, R.; Molander, S. In *Using DALY for Assessing Human Health Impacts of Conflict Minerals* Proceedings of the S-LCA conference, Pescara, Italy, 10-12 September, 2018.
26. UCDP (Uppsala Conflict Data Program) DR Congo (Zaire). Available online: http://ucdp.uu.se/#country/490 (accessed 10 January 2018)
27. Parsmo, R. The Blood Wedding Ring. Assessing the Life Cycle Lives Lost in Gold Jewelry Production. MSc thesis. Chalmers University of Technology, Gothenburg, Sweden, 2015.

28. USGS (U.S. Geological Survey) Historical Statistics for Mineral and Material Commodities in the United States. 2014 version. Data series 140. Available online: https://minerals.usgs.gov/minerals/pubs/historical-statistics/ (accessed on 10 January 2018)
29. KP (Kimberley Process) Democratic Republic Congo. Available online: https://www.kimberleyprocess.com/en/democratic-republic-congo (accessed on 10 January 2018)
30. Vahidi, E.; Zhao, F., Environmental life cycle assessment on the separation of rare earth oxides through solvent extraction. *J. Environ. Manage.* **2017,** 203, 255-263.
